# Fabrication of Bio-Nanocomposite Packaging Films with PVA, MMt Clay Nanoparticles, CNCs, and Essential Oils for the Postharvest Preservation of Sapota Fruits

**DOI:** 10.3390/polym15173589

**Published:** 2023-08-29

**Authors:** Senthamil Selvi Poongavanam, Vishnupriya Subramaniyan, Periyar Selvam Sellamuthu, Jayaramudu Jarugala, Emmanuel Rotimi Sadiku

**Affiliations:** 1 Department of Biotechnology, School of Bioengineering, SRM Institute of Science and Technology, Potheri, Kattankulathur, Chengalpattu 603203, Tamilnadu, India; sp1597@srmist.edu.in (S.S.P.); vs9820@srmist.edu.in (V.S.); 2 Department of Food Process Engineering, Postharvest Research Lab., School of Bioengineering, SRM Institute of Science and Technology, Potheri, Kattankulathur, Chengalpattu 603203, Tamilnadu, India; 3 Polymer and Petroleum Group, Material Sciences and Technology Division, CSIR-North East Institute of Science and Technology, Jorhat 785006, Assam, India; jayaramj@neist.res.in; 4 Institute of NanoEngineering Research (INER), Department of Chemical, Metallurgical and Materials Engineering, Pretoria West Campus, Tshwane University of Technology, Staatsartillerie Rd., Pretoria 0183, South Africa; sadikur@tut.ac.za

**Keywords:** natural fungicide, ajwain oil, oregano oil, garlic peel, decay index, shelf-life

## Abstract

Sapota is an important climacteric fruit with limited shelf life. A special system must be employed to extend the shelf life of sapota fruits. In the present study, polyvinyl alcohol (PVA) and montmorillonite clay (MMt)-based bio-nanocomposite films (BNFs) were integrated at various concentrations (2%, 4%, 6%, and 8%) into cellulose nanocrystals (CNCs), produced from garlic peels (GPs). The BNF loaded with 8% CNC has a better crystallinity index and mechanical properties than the other concentrations of CNC. Therefore, the 8% CNC-incorporated BNF (BNF-8) was selected for further packaging studies. The combined effect of BNF-8 with ajwain essential oil (AO) and oregano essential oil (OO) vapors and BNF-8 with carbendazim (commercial fungicide-CARB) were investigated. In this study, the BNF-based packagings are categorized into five types, viz: BNF+8% CNC (BNF-8), BNF-8+AO, BNF-8+OO, BNF-8+CARB and the non-packaged fruits (control). The shelf-life duration, antioxidant activity, firmness, decay index, and sensory quality were evaluated in order to identify the effectiveness of packaging treatment on sapota fruits. BNF-8+CARB, BNF-8+AO, and BNF-8+OO packaging extended the shelf life of sapota fruits to up to 12 days and maintained the overall physiochemical parameters and sensory qualities of the fruits. Therefore, the BNF-8+AO and BNF-8+OO packaging materials are appropriate alternatives to commercial fungicides for the preservation of sapota during postharvest storage.

## 1. Introduction

Sapota (*Manilkara zapota*) is a nutritious fruit that has been commended for its soft and delicious taste, with a pleasant aroma and a granular texture. Sapota fruits originated in the southern regions of Mexico, but it is now widely cultivated in sub-tropical and tropical nations. Sapota fruit’s postharvest shelf life is about 7 days. In addition to this, sapota fruits are susceptible to chilling injury when stockpiled at 10 °C. In tropical nations, the postharvest loss of sapota fruits is high due to the faster ripening, lack of appropriate storage facility, and rapid microbial decay effects [1]. Commercially, sapota fruits have been treated with Captan, Mancozeb, and Carbendizm in order to prolong their shelf life and protect them against postharvest diseases [2]. The use of chemical fungicides on sapota fruits has negatively impacted the environment and the health of consumers. Additionally, the use of chemical fungicides on fruits has negatively impacted the nutritional qualities of sapota fruits and considerably contributed to the development of disease-resistant postharvest pathogenic organisms [3,4]. Therefore, the sapota fruit’s postharvest quality should be preserved using appropriate postharvest techniques. One of the efficient postharvest methods to enhance the quality of sapota fruits is environment-friendly bio-nanocomposite packaging. Bio-nanocomposite packaging can increase the shelf life of fruits through its tendency to shield the fruits from water vapor permeability, gas transfer (O_2_ and CO_2_), and microbial attack [5]. Therefore, the greatest option to prevent the postharvest quality losses of fruits, especially sapota fruits, may be achieved through the bio-nanocomposite packaging route with natural fungicides, e.g., essential oils.

PVA is a synthetic biodegradable polymer. PVA polymers have high degrees of hydrolysis, molecular weights, tensile strength, adhesive strength, and water resistance attributes. Due to its biocompatibility, PVA has been used as a biopolymer in numerous disciplines, such as biomedical, pharmaceutical, and food packaging applications [6]. In recent years, bio-nanocomposite packaging has gained considerable attention from environmentalists and scientists due to its advantages, such as non-toxicity, biodegradability, and biocompatibility. Cellulose is a rich natural polymer, and it is found in numerous natural sources. Cellulose is used for a broad range of applications, and it plays a major role in the production of biodegradable packaging films [7,8]. Cellulose is a very readily available natural polysaccharide, and it is primarily found in agricultural wastes that have been widely employed in the production of cellulose nanocrystals (CNCs) [9]. Nanocelluloses, e.g., nanocrystals and nanofibers, are a more valuable class of material produced using mechanical and chemical processing methods of cellulose from the natural native source. Cellulose nanocrystals (CNCs) have attracted a lot of interest, especially in the field of nanotechnology, owing to their excellent mechanical strength, low cost, environmental friendliness and their excellent thermal and barrier properties [10]. CNCs are obtained from many agricultural wastes, such as rice straw and husk, wheat, areca and grain straw, kenaf, sugarcane bagasse, cotton waste, and fruit and vegetable peels [11]. Garlic (*Allium sativum* L.) has been extensively utilized in several parts of the world due to its culinary and medicinal properties. Garlic peel (GP) is one of these potential materials, and it is an underused cellulose source because the utilization of garlic among the world population is increasing. However, GP has a limited possibility of recycling [12]. Therefore, GP has a comparatively high cellulose content, and it is a desirable material for utilization in the fabrication of bio-composites [13]. Only a small number of studies have been published on CNC extraction from GP and the use of CNCs as strengthening elements in many polymeric mixtures [14]. Similar to CNCs, montmorillonite (MMt) clay has a great strengthening property, and it is the most popular natural clay that has been successfully employed in many nanocomposite production methods [15]. MMt is a cost-efficient and non-toxic clay mineral that interacts with a variety of biomolecules, including polymers, and it is a member of the phyllosilicates family [16]. MMt clays have numerous desirable applications due to their superior mechanical and thermal properties compared with pure polymers [17,18]. The addition of MMt clay into the polymer matrix aids in enhancing the packaging characteristics of the polymer. Therefore, it is gaining significant attention among the packaging industries owing to its beneficial features [19]. Additionally, MMt clay is hydrophilic, and it can form stable suspensions in water, which makes it easier for it to dissolve in a polymer matrix [20]. Furthermore, essential oils (EOs), e.g., oregano (OO) and ajwain (AO) oil, have been used in packaging materials to prevent microbial attack on fresh produce, owing to their antimicrobial nature and they have the ability to impede the activity of postharvest pathogenic organisms on fruits [21]. Therefore, this study aims to isolate CNCs from GP and incorporate the various concentrations of CNCs into the PVA and MMt clay mixture and to evaluate the structural (crystallinity), thermal stability, hydrophobicity, water vapor permeability, oxygen transfer rate and mechanical characteristics of the bio-nanocomposite-based films. Together with this, the effect of AO and OO vapors on the sapota fruits in bio-nanocomposite packaging films were also evaluated.

## 2. Materials and Methods

### 2.1. Extraction of CNC from GP

Garlic peels were collected from the local market in Chennai, Tamilnadu State, India. Garlic skins (wastes) were washed with deionized water. Then, the garlic peels (GPs) were subjected to alkaline treatment (5% sodium hydroxide solution) for three hours at 80 °C, and this process was repeated three times. After the treatment process, bleaching treatment was carried out with a solution of 1.7% *w*/*v* sodium chloride in an acetate buffer for 4 h at 80 °C in order to eliminate the residual lignin present. Subsequently, the acquired fibers were washed, filtered, and air-dried. To synthesize CNCs, GP fibers were subjected to acid hydrolysis in 65% sulphuric acid under continuous stirring at 45 °C for 40 min. The reaction was interrupted by diluting the dispersion with cold distilled water, and excessive sulfuric acid was removed by the repeated centrifugation process at 10,000 rpm for 10 min at 10 °C. The resultant aqueous dispersion was dialyzed against distilled water with a cellulose membrane until it reached a neutral pH. Then, for the dispersion of the CNC, the final mixture was sonicated for 45 min and finally stored at 4 °C for future use [14,15].

### 2.2. Preparation of Bio-Nanocomposite Films

Polyvinyl alcohol (PVA)-based bio-nanocomposite films (BNFs), which contain montmorillonite clay (MMt) and CNC, were prepared using a solvent casting procedure [20,22]. PVA (5%) was mixed with distilled water at 90 °C for 1 h using a magnetic stirrer until the PVA was finely blended in the solution. The 20 wt% of MMt clay was employed, based on the weight percentage of PVA, and it was dissolved in distilled water with persistent stirring and blended with the PVA solution. Then, polyethylene glycol-400 (PEG-0.5 mL) was added as a plasticizer and the PEG acted as the cross-linking compound. Thereafter, different concentrations of CNC (2%, 4%, 6% and 8%), obtained from GP, were added in to the PVA/MMt solution (BNF). The PVA/CNC/MMt mixture was cast in the Petri dish and dried at 45 °C for 24 h. For comparison purposes, the neat PVA films and the PVA/MMt clay-based films were also cast. The BNFs were coded thus: neat PVA, PVA+MMt, PVA+MMt+2%CNC, PVA+MMt+4%CNC, PVA+MMt+6%CNC, and PVA+MMt+8%CNC. Similarly, the BNF solution, with optimum CNC concentration was cast in a rectangular glass plate for the fruit packaging studies.

### 2.3. Scanning Electron Microscope

The morphologies of the BNFs (neat PVA, PVA+MMt, PVA+MMt+2%CNCs, PVA+MMt+4%CNCs, PVA+MMt+6%CNCs, and PVA+MMt+8%CNCs) and GP were examined using an Apero Scanning Electron Microscope (SEM) [Thermo Scientific, Waltham, MA, USA]. The samples were mounted on the stubs of the SEM platform/sample holder. The fiber, CNC, and BNF films were sputtered/coated with gold (to improve electron conductivity in the tube) through an ion coater and examined at an accelerating voltage of 10 kV.

### 2.4. X-ray Diffraction

The diffraction patterns of the BNF samples were obtained using a PANalytical, Xpert3 Diffractometer (Malvern, UK), which is equipped with the CRISP technology and enabled by the 2nd generation PreFIX technology, pneumatic shutters and with beams of 59 attenuators and Cu-K_α_ radiation (wavelength, λ = 1.54 Å) and generated at 45 kV below a current of 40 mA using a scan rate of 2θ = 10 min^−1^ in the 2θ ranged between 10° and 90°. The 2θ reflection angle was used to acquire the diffraction patterns.

The following equation was used to calculate the crystallinity index (CrI)

CrI=I200−Iam/I200

where Iam is the lowest intensity related to the amorphous structure, and I200 is the highest intensity of the diffraction peak.

### 2.5. Mechanical Properties

A universal tensile testing device (BIE, model TKG-EC- 2500, Kolkata, India) was used to determine the films’ tensile strength, elongation-at-break, and elastic modulus. After setting the gauge length to 25 mm and the crosshead speed to 10 mm/min, the film samples with dimensions of 6 × 1.5 cm were subjected to a tensile strength test. This is for the purpose of determining the mechanical properties, including the stress–strain behaviors of the samples.

### 2.6. UV Visible Spectroscopy

The UV-visible spectra of the BNFs were measured using a German-made Analytik Jena UV-visible/DRS spectrophotometer (SPECORD 210 PLUS, Jena, Germany). The sample’s transparency was examined in the wavelength range between 200 and 900 nm.

### 2.7. Thermogravimetric Analysis

A thermogravimetric analyzer (TGA) Perkin Elmer STA6000 (Waltham, MA, USA) was used to analyze the thermal stability of the BNFs. To determine the decomposition peak values and transition temperature, the film samples were subjected to heating at a rate of 10 °C per minute in a nitrogen environment (140 mL/min) between 50° and 800 °C.

### 2.8. Contact Angle

Static contact angle measurements were used to examine the hydrophobic nature of the neat PVA, PVA+MMt, PVA+MMt+2%CNCs, PVA+MMt+4%CNCs, PVA+MMt+6%CNCs, and PVA+MMt+8%CNCs films. The water contact angles were measured using the Sessile drop method, with a contact angle goniometer KYOWA DMS-401, half-angle technique fitting, and interface Measurement and Analysis System (FAMAS) Add-in software, version 5.0.30 (Saitama, Japan). The measurement was performed using water and air as the probe liquid. The static contact angle was measured on three distinct film sites.

### 2.9. Atomic Force Microscopy (AFM)

For the AFM imaging, an SPM-9500 (Shimadzu Co. Ltd., Kyoto, Japan) and a D3000 (Digital Instruments, Santa Barbara, CA, USA) were employed. A 25 mm^2^ each of the neat PVA, PVA+MMt, PVA+MMt+2%CNCs, PVA+MMt+4%CNCs, PVA+MMt+6%CNCs, and PVA+MMt+8%CNCs films was glued on double-sided tape onto the sample stage. Then, a 120 m scanner from Digital Instruments and a Model TESP etched silicon probe with a resonance frequency of about 270 kHz. The imaging duration for each frame was estimated to be between 4 and 10 min, based on the scan rate range of 0.5 to 1 Hz. In order to avoid damaging the device, the set line voltage was changed to the lowest permissible voltage, between 1.6 and 2.5 V, and the images of the films were recorded at 512 × 512 pixels.

### 2.10. Water Vapor Permeability

The crucial factor that affects the food packaging films’ commercial success is water vapor permeability (WVP). The ASTM E96 technique was used to measure the optimized BNF-8 film. In general, the WVP is the transmission of vapor over a unit surface on an even material with a unit thickness when there is a unit vapor pressure differential between the two surfaces [23]. A Teflon tape was used to seal a permeation cell containing 2 mL of water with the sample film, and the desiccator was set to 20 °C, 0% relative humidity (RH), and 0 kPa vapor pressure. For 24 h, the decrease in the weight of the permeation cell, caused by the passage of water vapor to the desiccator, was measured at regular intervals. The WVP value was calculated using the equation below:

wx/A∆P=WVP×t

Herein, x represents the BNF’s thickness in millimeters, A: area of permeation in square meters, P: partial pressure difference in kPa, *t*: time in days, and w: mass loss in grams [24].

### 2.11. Oxygen Transfer Rate

The oxygen transfer rate (OTR), measured under standard test circumstances and given as cm^3^ (STP)/m^2^ day, is the constant flow of O_2_ gas through the film’s surface per unit area and per minute over a unit partial pressure that differs on the two sides of the material. This test was carried out in accordance with the ISO 15105 Part-1 [25], which outlines OTR through a one-layer film under divergent pressure. PVA+MMt+CNC 8% film samples were secured in an oxygen-free diffusion cell for this study. Subsequently, 99.9% pure oxygen was permitted to flow into the cell across the film and out on the other side of the film. A coulometric detector at a stable flow rate observed the oxygen seeping over the BNF in relation to the oxygen pressure gradient.

### 2.12. Experimental Design for Packaging Studies

Based on the characterization studies, the bio-nanocomposite films, which showed better characteristics, were taken for further studies. Furthermore, 66.67 µL/L of oregano and ajwain essential oils, integrated with bio-nanocomposite films, were studied. The essential oil concentration was fixed according to the concentration of the essential oil required to inhibit sapota post-harvest pathogens. Therefore, the oregano and ajwain essential oils used in this study are based on our in vitro antifungal study, which has yet to be published and is not reported. In order to compare with commercial fungicides, the sapota fruits were dipped into carbendazim or CARB (500 µL/liter) and allowed to dry; thereafter, the fruits were packaged with BNF-8. Then, a 66.67 µL of AO and OO-loaded Whatman filter paper sheet was placed separately inside the BNF. The oil-loaded BNFs were categorized as BNF-8 +AO and BNF-8+OO. The non-packaged fruits served as the control samples.

### 2.13. Decay Incidence

According to the Khaliq et al. procedure, the decay incidence was investigated [26]. Visible fungal growth on the sapota fruits was used to determine the decay rate or incidence or index. Fruit decay was calculated using the equation below:

Decay incidence (%)=100×∑AB/CD

where A is the decay level, B is the number of fruits at this level, C is the total number of fruits under study, and D is the highest level of decay.

### 2.14. Fruit Firmness

The fruit’s firmness indicates the fruit ripening process. The ripened fruits have less firmness than unripened fruits. Therefore, the texture analyzer (TA-XT-plus, Godalming, UK) was used to measure the firmness of the sapota fruits. A 2 mm cylindrical probe was used to puncture the equatorial area of the sapota fruits, and the results are represented in Newton (N), which denotes the force needed to puncture a hole in the fruits.

### 2.15. Antioxidant Activity

The antioxidant or radical scavenging activity was determined following the Perumal et al. procedure, with slight changes [27]. The 2,2-diphenyl-1-picrylhydrazyl (DPPH) assay was used to assess the radical scavenging activity. Methanol, in a water mixture ratio (60:40), was utilized to obtain samples of 2 g of the sapota fruit. Then, a diphenyl-1-picryhydrazyl (DPPH) solution of 250 µL was added to the micro-titer reader plate. The samples (30 µL) were gradually mixed and held at room temperature in a dark atmosphere for 20 min, and the absorbance was measured at a wavelength of 517 nm. The DPPH radical scavenging activity was calculated using the following equation:

DPPH scavenging activity (%)=[(control−sample)/control]×100

### 2.16. Sensory Analysis

A hedonic scale of 1 to 9 was used to qualitatively score the sensory qualities of the sapota fruits. The hedonic scale between 1 and 3 is considered extremely bad, between 3 and 5 is rated as neither likely nor unlikely, between 5 and 7 is rated as fair, and between 7 and 9 is rated as very good. The flavor, texture, taste, and overall acceptability of the packaged and the non-packaged fruits were measured by sensory analysis. These ratings were conducted by a group of thirty panelists who were from diverse ages and genders from the School of Bioengineering, SRMIST, Kattankulathur, Tamil Nadu, India. A random sample of the fruit from each treatment was given to each panelist.

### 2.17. Statistical Analysis

Data obtained from these investigations were expressed as mean ± standard deviation. Duncan’s test (*p* < 0.05) was performed in order to examine the statistical variance after a one-way ANOVA test using the Statistical Package for the Social Sciences (IBM SPSS, V23, Chicago, IL, USA) software.

## 3. Results and Discussion

### 3.1. Morphological Analysis of BNFs

**Figure 1:**
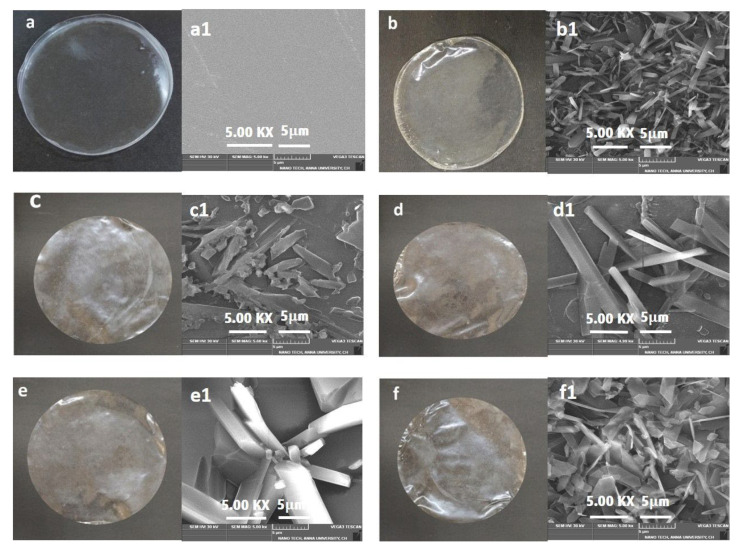
Photographs of bio-nanocomposite films: (**a**) Neat PVA, (**b**) PVA+MMt clay, (**c**) PVA+MMt clay+CNC 2%, (**d**) PVA+MMt clay+CNC 4%, (**e**) PVA+MMt clay+CNC 6% and (**f**) PVA+MMt clay+CNC 8%. SEM images of bio-nanocomposite films: (**a1**) Neat PVA, (**b1**) PVA+MMt clay, (**c1**) PVA+MMt clay+CNC 2%, (**d1**) PVA+MMt clay+CNC 4%, (**e1**) PVA+MMt clay+CNC 6% and (**f1**) PVA+MMt clay+CNC 8%.

The surfaces of the neat PVA, PVA+MMt+2% CNC, PVA+MMt+4% CNC, PVA+MMt+6% CNC, and PVA+MMt+8% CNC films were subjected to SEM examination in order to assess the micro-structural changes brought about by the addition of MMt clay+PVA and MMt clay+PVA/CNCs (2–8%) films. The digital and SEM images of BNFS are represented in Figure 1. SEM image of the neat PVA shows that it had a clean, homogenous surface that is free from pores, fissures, and abnormalities (Figure 1(a1)). The SEM image of the PVA/MMt clay film (Figure 1(b1)) shows some degree of heterogeneity owing to the agglomeration of clay particles in some regions of the films. The addition of MMt nanoclay to the PVA matrix made the surface rougher than the neat PVA, and some studies have reported that MMt clays have the capability to agglomerate at greater concentrations, which led to an uneven distribution in the PVA matrix [19,20]. The inclusion of CNCs to the MMt clay-PVA film resulted in an evenly distributed MMt clay and CNCs (Figure 1(c1–f1)). The outcome of this study showed that the PVA/MMt clay and the CNC polymer were compatible with one another, which resulted in the production of homogeneous bio-nanocomposite films [PVA+MMt+CNC]. In addition to these findings, numerous reports have claimed that CNCs have even distribution in the films; however, an increasing percentage of CNC in the polymer matrix displayed rougher features with several aggregations than the neat PVA [21,28,29]. Therefore, the inclusions of MMt clay and CNC in the films resulted in a rougher surface than in neat PVA and MMt clay-based films.

### 3.2. X-ray Diffraction of BNFs

**Figure 2:**
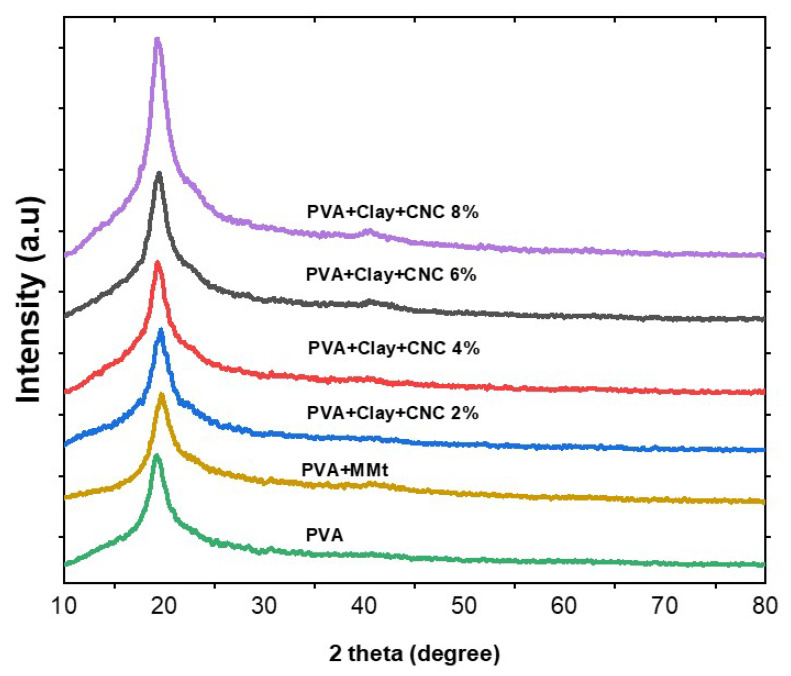
X-ray diffraction of bio-nanocomposite films with different CNC concentrations.

X-ray diffraction (XRD) analysis was carried out in order to ascertain the crystallinity index of the films. The XRD patterns of the neat PVA, PVA+MMt, PVA+MMt+CNC 2%, PVA+MMt+CNC 4%, PVA+MMt+CNC 6%, and PVA+MMt+CNC 8%, are presented in Figure 2. The PVA-based films containing CNCs (2 to 8%) and MMt clay showed a considerable peak in the 2θ range between 18.12 and 22.16°. The outcome of this study shows that the: neat PVA, MMt+PVA, and MMt+PVA+CNC 2% have highly similar crystallinity patterns. The incorporation of MMt, 2% of CNCs did not significantly affect the crystallinity patterns, whereas increasing the CNC content increased the crystallinity of the films: PVA+MMt+CNC 4% resulted in a crystal plane at 2θ = 19.18°, PVA+MMt+CNC 6% at 2θ = 21.42°, and PVA+MMt+CNC 8% at 2θ = 22.16°. Therefore, the intensity of the diffraction peaks increased as the amount of the CNC in the films increased, which suggests that the CNC was successfully incorporated into the films. Identical to our results, Gupta et al. [30] and Perumal et al. [29] have reported that increasing the amount or concentration of CNC improved the crystallinity pattern. In addition, these studies suggest that the presence of cellulose in the films, in the form of CNCs, is responsible for the improved crystallinity.

### 3.3. Tensile Strength of the BNFs

**Figure 3:**
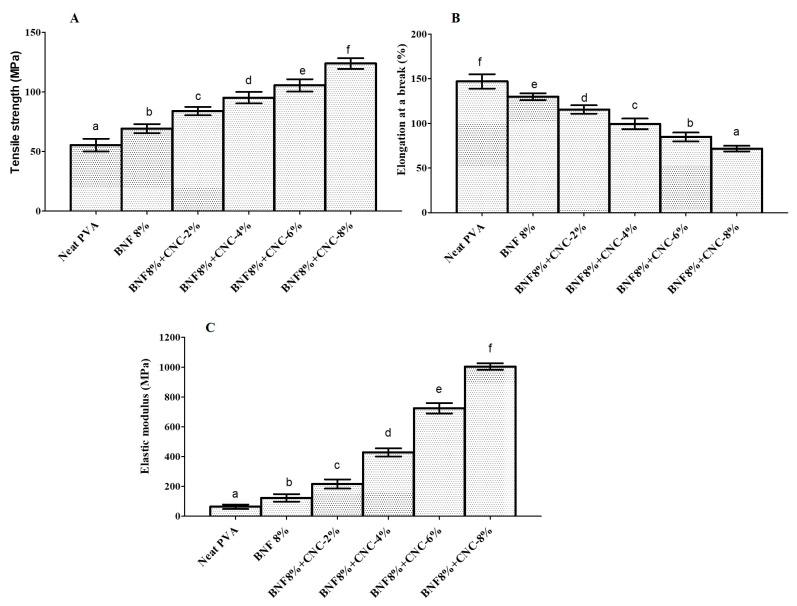
(**A**) Tensile strength (**B**), Elongation-at-break, and (**C**) Elastic modulus of the bio-nanocomposite films. The lower-case letters indicate the mean values with significant differences between the different compositions of biocomposite films according to Duncan’s test (*p* < 0.05).

The BNF tensile strengths, elongation-at-break, and elasticity modulus are significantly increased upon the addition of CNCs. As shown in Figure 3A, the tensile strength increased progressively, from 55.1 MPa (neat PVA) to 123.5 MPa [PVA+MMt+CNC 8%]. Similarly, the elongation-at-break reduced from 147% to 71.3%, while Young’s modulus, or modulus of elasticity, abruptly inclined from 63.3 MPa to 1312 MPa, respectively, and these observations are presented in Figure 3B,C. The findings of this study are consistently identical to research studies conducted by Wang et al. [31] and Perumal et al. [32], in which a rise in the tensile strength and a reduction in the elongation of the films were reported with an increase in the concentration of CNCs. The reduction in elongation might be due to the intense interaction of the higher concentration of CNCs, which resulted in film hardening, and the decreased concentration of CNCs, led to brittle films. According to this observation, the addition of CNC 8% improved the mechanical properties of the BNF. CNC and MMt clay inclusions played an important role in the mechanical properties of the films, and this is related to the uniform distribution of CNCs and the positive interfacial contact of CNCs and MMt clay with the PVA matrix. Singh et al. [33] and Achaby et al. [34] also, respectively, reported the fact that PVA films recorded increases in the tensile strengths of 5% and 8% with increases in CNC contents. In addition to this, many studies have shown that reinforcing the matrix with MMt clay and with CNC enhanced the tensile strength of the films [27,35,36,37,38]. Based on the morphological and mechanical studies, the PVA+MMt+CNC 8% bio-nanocomposite was selected for the packaging studies.

### 3.4. UV Transparency of Films

**Figure 4:**
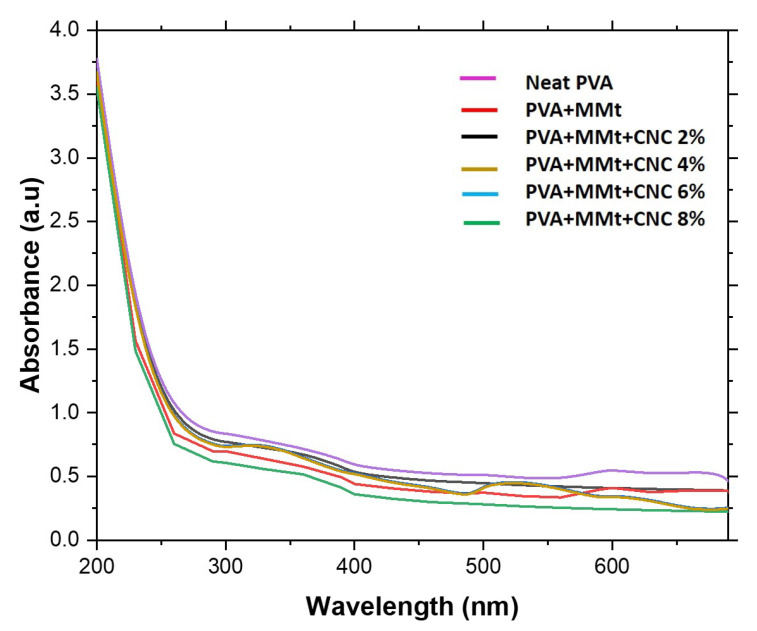
UV-visible absorbance spectra of the packaging films (neat PVA, PVA+MMt, PVA+MMt+CNC 2%, PVA+MMt+CNC 4%, PVA+MMt+CNC 6% and PVA+MMt+CNC 8% films).

Blocking the harmful wavelength of light is a crucial feature in packing material since UV light energy can lead to oxidation reactions in food that may result in off-flavor and odor. Figure 4 displays the absorption spectra of neat PVA, PVA+MMt, PVA+MMt+CNC 2%, PVA+MMt+CNC 4%, PVA+MMt+CNC 6%, and PVA+MMt+CNC 8% films. All the composition of packaging films has highly absorbed UV light between 200 and 250 nm, and the absorption of UV light has substantially decreased in the UV region (200–400 nm). However, packaging films containing MMt clay-PVA-CNC 8% have minimally absorbed UV light compared with other films. Therefore, MMt clay-PVA-CNC 8% has better UV barrier properties when compared to the other films. This strongly indicates the fact that PVA+MMt+CNC 8% films can function as UV light barriers, and this can prevent the deterioration of food from UV light. Similarly, transparency (visible) is a necessary property of biocomposites for packaging applications since it allows for the visual inspection of the packaged goods. The PVA films have higher transparency value (400–700 nm) than other films, next to the PVA films, PVA+MMt, PVA+MMt+CNC 2%, PVA+MMt+CNC 4%, PVA+MMt+CNC 6% has higher transparency rate. PVA+MMt+CNC 8% has lower transparency than the other films, and the transparency of the packaging film was slightly influenced by adding CNC-8%. Although PVA+MMt+CNC 8% film has lower transparency, it did not affect the visual view of the food product. Similar to our study, Yadav et al. [39] found that increasing the concentration of CNC in PVA could effectively act as a UV barrier. In addition, according to Zhang et al. [40], CNC reinforcement reduced UV absorption in PVC thin films. Sukyai et al. [16] reported that the integration of MMt clay into a food packaging film reduced the transmission of UV light in the film, and it served as a UV barrier. In addition, the neat PVA+MMT+CNC 8% film had a lower transmittance value than the PVA film, which resulted in a lower transmittance, and hence, the transparency of the PVA matrix was slightly influenced upon its mixing with CNC and MMt. However, the lower transparency in the PVA+MMt+CNC 8% films did not affect the visual inspection of packaged goods.

### 3.5. Contact Angle of BNFs

**Figure 5:**
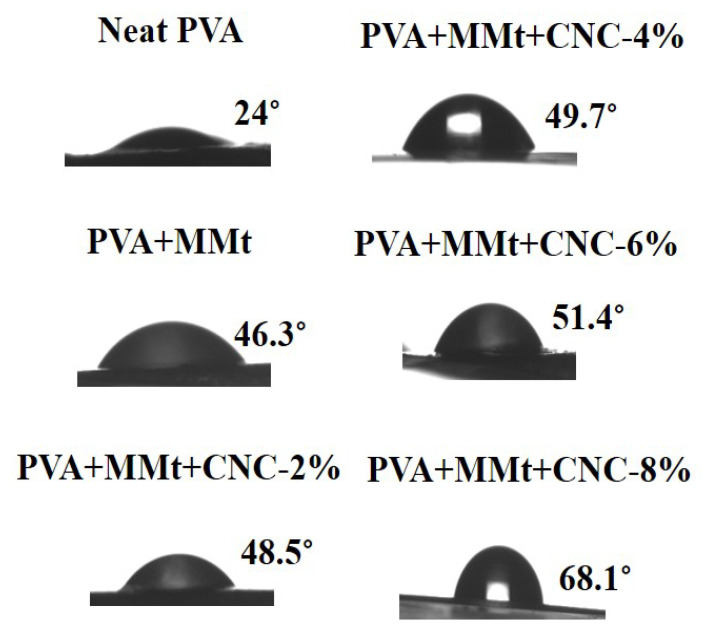
Contact angle of neat PVA, PVA+MMt, PVA+MMt+CNC 2%, PVA+MMt+CNC 4%, PVA+MMt+CNC 6% and PVA+MMt+CNC 8% films.

The contact angle of the film samples is displayed in Figure 5. A contact angle of 24° was visible on the clean/neat PVA film. This phenomenon illustrates the PVA matrix’s hydrophilic and wettable properties. MMt was included in the PVA matrix, which changes the contact angle from 24° to 46.3°, i.e., it was dramatically raised for the PVA-MMt matrix. In addition, after the CNC fillers (2, 4, 6, and 8%) were added to the PVA-MMt matrix, the nanocomposites’ contact angles increased. However, the 2, 4, and 6% concentrations of CNC showed minimal impact on the improvement of hydrophobicity. The films, which integrated 2 to 6% CNC contents, had their contact angles increased from 48.5° to 51.4°. When the CNC content was increased from 2 to 8%, the hydrophobicity of the film increased drastically. The contact angle of the PVA+MMt+CNC 8% film recorded a 68.1° value, which is the highest among all the samples. Based on the results of this study, the PVA/MMt/CNC-8 sample recorded the best hydrophobicity of all the films. Identical outcomes demonstrated that the hydrophobicity of nanocellulose and alginate-based films increased when CNC was added [41]. He et al. [42] investigated the hydrophobicity of carboxy methyl cellulose and MMt-based films, and the results showed that the incorporation of MMT clay into the carboxy methyl cellulose matrix significantly enhanced the hydrophobic nature of the films. Therefore, the combination of MMt and CNC-8 enhanced the hydrophobicity of the films.

### 3.6. Atomic Force Microscope

**Figure 6:**
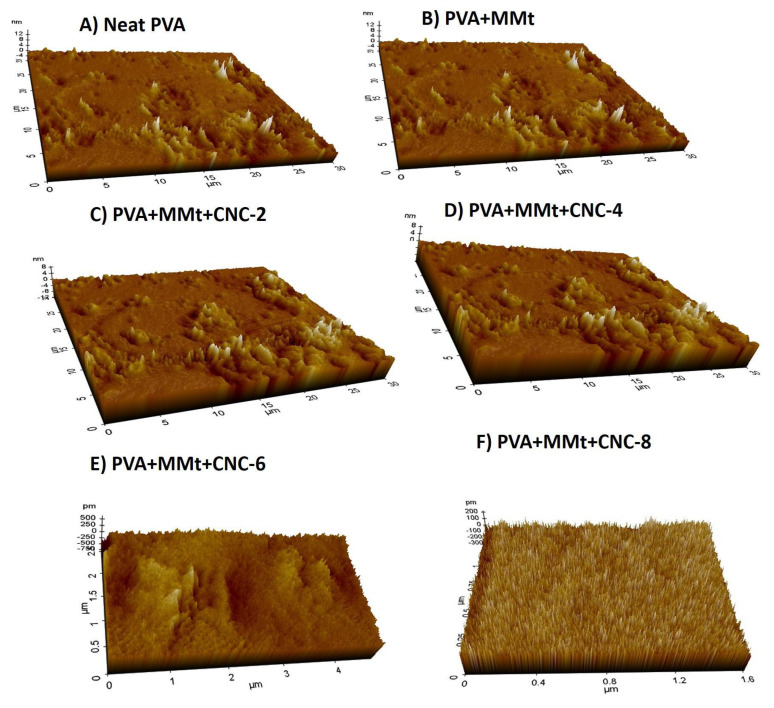
Three-dimensional AFM topographic pictures of films.

In order to have a good examination of the topography of films, the atomic force microscopy (AFM) technique is a potent tool for providing qualitative and quantitative information on the surface morphology of films at the nanoscale. AFM two and tri-dimensional models are displayed in Figure S1 and Figure 6. The control film had a uniform, compact, smooth, and cohesive internal structure. This is the result of the existence of a homogenous and ordered-phase network structure. In comparison to the control films, the surface of the PVA/MMt-based films exhibited a slightly rougher appearance. Furthermore, the addition of CNC in the packaging films increased the roughness of the films. Additionally, the internal structure and film surfaces were impacted by the addition of MMT and CNC, and these inclusions led to the loss of smooth features in the films. AFM roughness measurements are frequently related to contact angle analysis. Many investigations stated that an increase in surface heterogenicity or roughness increases the hydrophobic nature of the film. In agreement with these investigations, PVA+MMt+CNC 8% has higher surface roughness and hydrophobicity than other films [43,44]. Similar trends have been identified in the SEM images of the films; the incorporation of CNC and MMT caused the coarse surface areas. Alexandre et al. [45] observed a similar pattern of behavior. This phenomenon is believed to have occurred due to the formation of agglomerations, which led to less homogeneous structures, or as a result of material elutriation to the surface during the drying process. Similarly, the surface roughness of the films changed depending on the addition of CNC content. The investigation showed that the integration of CNC into films resulted in the roughness observed on the films surfaces [46].

### 3.7. Thermogravimetric Analysis

**Figure 7:**
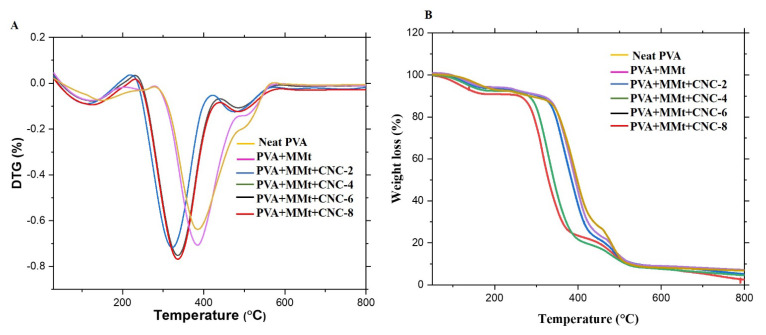
(**A**) Thermal characteristics of the bio-nanocomposite films and (**B**) DTG curve of the bio-nanocomposite films.

The TGA (Thermogravimetric analysis) and DTG (Derivative thermogravimetric) plots, shown in Figure 7A,B, were used to analyze the BNFs thermal stability, following the addition of MMt clay and CNCs. From each of these plots, it can be seen that the films degraded in stages across a wide temperature range between 50 °C and 800 °C. The BNF degraded in two stages, and the BNFs initial thermal deterioration stage took place between 50 °C and 150 °C, which was caused by the deterioration of the low molecular weight compounds and moisture evaporation. The second stage of the BNF degradation occurred from 200 to 300 °C. Significant weight losses for all the samples can be observed, and this observation is represented in Figure 7. The side chain breakdowns of PVA, glycerol, and CNC are the causes of weight loss in the first and second stages. The major chains of PVA degraded at a higher rate in the third stage of thermal degradation, which occurred between 325 °C and 450 °C. This is a result of the cyclization reaction and the continuous removal of the remaining acetate groups. The PVA+MMt+CNC 8% composite film, with a T_max_ of 400 °C, demonstrated greater thermal stability than other films, notably in the temperature range between 400 °C and 450 °C. The PVA+MMt+CNC 8% recorded higher thermal degradation than the PVA+MMt+CNC 2%, PVA+MMt+CNC 4% and PVA+MMt+CNC 6% films. The thermal degradation profiles of all the films are similar. However, the thermal degradation of PVA+MMt+CNC 8% slightly decreased compared with the other films. This is believed to be the result of the creation of a denser network, brought about by the addition of a high amount of CNC to the PVA/MMt mixture. In addition, MMt clay and CNC to the PVA increased the tensile strength, elongation, hydrophobicity, and crystallinity of the films. Based on this study, it is obvious that the MMt clay and the rising CNC concentration moderately enhanced the thermal stability of the films. Similar outcomes were reported by Kang et al. [47], who found that the inclusion of CNC-8%, derived from wheat bran, significantly enhanced the thermal stability of the biocomposite films under study. Oun et al. [48] observed that the thermal stability of films made by the addition of CNC to the polyvinyl alcohol matrix was marginally improved. Similar to our results, Lizundia et al. [49] proved that the addition of CNC slightly improved the thermal stability of films under investigation; however, it did not majorly influence the thermal stability of films.

### 3.8. Barrier Properties

In this investigation, the film with MMt and 8% CNC was found to have superior hydrophobic nature and biophysical, mechanical, and thermal properties than all other films. Therefore, the barrier property was investigated for the PVA+MMt+CNC 8% film. 

#### Water Vapor Permeability (WVP) and Oxygen Transmission Rate (OTR)

The shelf life and quality of the packaged food product were determined by the water vapor barrier property of a food packaging film (of a specific thickness). A low WVP (high water vapor barrier) is preferred for perishable and extremely perishable food products. The water vapor permeability of PVA+MMt+CNC 8% film with 0.5 ± 0.05 mm thickness recorded a WVP of 6.97 g/m^2^/day. Furthermore, the OTR for the PVA+MMt+CNC 8% film with a thickness of 0.5 ± 0.05 mm was 16.602 cm^3^/m^2^/day at 0.1 MPa. For the majority of food products, OTR values of food packaging films that range between 0.1 and 30 cm^3^/m^2^/day are highly desirable.

WVP is a significant and desirable characteristic to be considered in food packaging films. The addition of MMt and CNC-8% has increased the hydrophobic nature of the BNF-8 films. Therefore, PVA+MMt+CNC 8% film has low WVP, and it is considered suitable for packaging highly perishable fresh produce. In accordance with similar investigations, packaging films incorporated with the MMt clay have improved WVP and OTR. Enescu et al. [50] reported that the incorporation of MMt and CNC remarkably decreased the OTR and WVP values. In this study, the 8% CNC and MMt inclusions were well dispersed in the PVA matrix, and it blocked the permeation of water vapor and oxygen in the packaging films. In addition, the PVA+MMt+CNC 8% film provided a good barrier to OTR and WVP.

### 3.9. Packaging Studies

The characterization studies showed that PVA+MMt+8%CNC [BNF 8%] film exhibited better results. Therefore, the BNF 8% films were utilized for the packaging studies on sapota fruits. Hence, the sapota fruits were packaged with BNF8%, BNF8%+CARB, BNF8%+AO, and BNF8%+OO essential oil vapors in order to determine the shelf life where the non-packaged fruits acted as the control. The sapota fruits were placed inside the packaging films, viz: BNF 8%, BNF 8%+CARB, BNF 8%+AO, and BNF 8%+OO, and then, the packaging film was sealed. The packaged fruits were kept at ambient temperature in order to evaluate the shelf life and fruit quality.

#### 3.9.1. Decay Incidence

**Figure 8:**
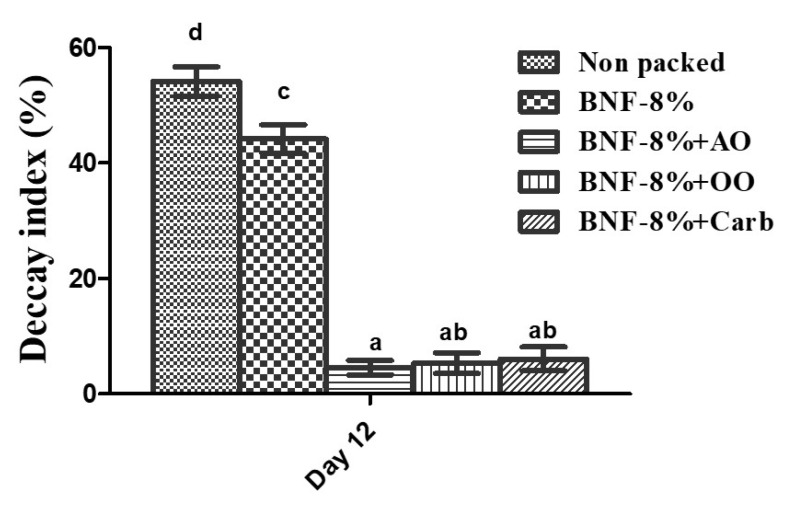
Decay incidence of sapodilla fruit during storage for 12 days. The lower-case letters indicate the mean values with significant differences between the packaging of fruits according to Duncan’s test (*p* < 0.05).

The decay incidence of sapota fruit grew gradually and reached a high level at the end of the storage period. Yet, the bio-nanocomposite film, containing oregano and ajwain essential oils, lowered the decay incidence of the sapota fruits throughout the storage time. In addition, the decay incidence of the fruits treated with carbendazim is similar to that of the BN 8%+OO and BNF 8%+AO packaged fruits. However, the BNF 8% and the non-packaged fruits recorded higher decay incidence at the end of the storage period, as depicted in Figure 8. Throughout the storage period, the non-packaged fruits had a higher visible deterioration frequency than those packaged with the BN 8%+OO and BNF 8%+AO, and BNF+carbendizm films. Sapota fruits are sensitive to postharvest microorganisms, and subsequently, they started to decay gradually as the disease resistance ability decreased during the senescence stage. Many investigation outcomes have suggested that the combination of bio-nanocomposites with essential oil significantly decreased postharvest infections in various fresh products. In addition, many investigations have already proved that OO and AO vapors have antimicrobial effects against numerous postharvest pathogenic organisms [30,37,38]. Consequently, cinnamon EO vapors, in modified atmospheric packaging, controlled the spoilage in cherry tomato and pepper fruits [31]. Perumal and co-workers reported that a modified atmosphere packaging, associated with cinnamon, thyme, and clove EO vapors, exhibited an extended shelf life of two mango varieties (i.e., Totapuri and Banganapalli), and it also effectively reduced the postharvest decay index in the above-mentioned two mango varieties [27]. Similarly, a modified atmosphere packaging with thyme EO vapors diminished the anthracnose disease incidence in avocado fruits [32]. The average shelf life of the sapota fruits is between 7 and 9 days, whereas the BN 8%+OO and BNF 8%+AO, and BN 8%+carbendazim-packaged sapota fruits, improved the sapota fruit’s shelf life to 12 days.

#### 3.9.2. Fruit Firmness

**Figure 9:**
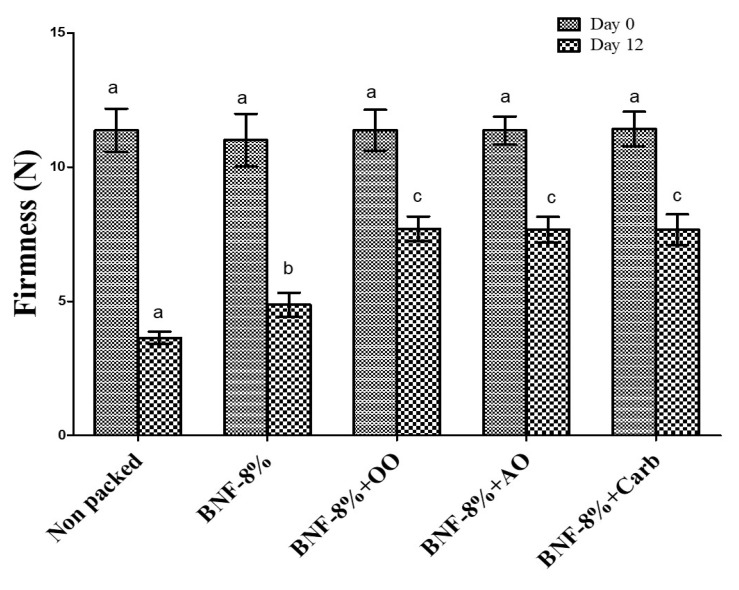
Firmness of treated sapota fruit from Day 0 to Day 12. The lower-case letters indicate the mean values with significant differences between the packaging of fruits according to Duncan’s test (*p* < 0.05).

Fruit firmness or hardness is the major quality attribute that influences the consumers’ acceptability of fresh fruit produce. Over the period of storage, the hardness of sapota fruits was reduced progressively in all the samples studied, and this phenomenon is represented in Figure 9. During the 0th day, all fruits maintained similar firmness. However, after 12 days of storage, the sapota fruit packaged with BNF 8%+AO and BNF 8%+OO recorded considerably greater firmness than the BNF and non-packaged fruits. Similar to our previous studies, mango fruits packaged using modified atmosphere packaging material with thyme essential oil inclusion maintained the firmness of the fruits. Bangar et al. suggested that Pueraria montana+Clove bud essential oil+CNCs+Peal millet starch maintained the firmness of red grapes at 5 °C for 15 days [33]. In another study, Zataria multiflora essential oil, incorporated in a PVA matrix, recorded a 27.2% firmness of strawberry fruits by up to 15 days of storage [34]. Furthermore, clove essential oil, integrated with active packaging materials, prolonged the firmness and shelf life of table grapes at 13 °C for 21 days [51]. Therefore, based on the reports, EO incorporation in active packaging materials protects the loss of firmness in fruits. In agreement with this, the use of BNF 8%+AO and BNF 8%+OO in the present study maintained the sapota fruit firmness until the 12th day of storage. In addition, the ripening of sapota fruits with the use of BNF 8%+AO and BNF 8%+OO was the slowest, even slower than the non-packaged fruits.

#### 3.9.3. Antioxidant Activity of Sapota Fruits Packed in the BNF

**Figure 10:**
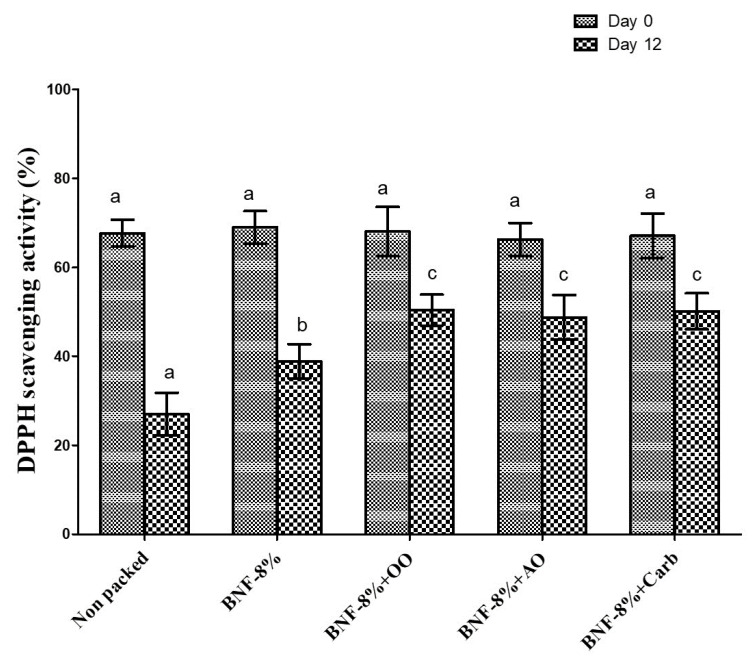
Antioxidant activity of treated sapota fruit during storage at ambient temperature between Day 0 and Day 12 of storage. The lower-case letters indicate the mean values with significant differences between the packaging of fruits according to Duncan’s test (*p* < 0.05).

Antioxidant activity is the major key factor in the determination of the quality of sapota fruits. The effect of the antioxidant activity on the various combinations of packaging films is represented in Figure 10. During the 0th day of storage, the antioxidant activity was constant for all the treated fruits. However, the antioxidant activity was decreased at the end of the storage period. On the 12th day of the storage period, the maximum antioxidant activity was observed in the fruits packaged with BNF-8%+AO and BNF-8%+OO and the carbendazim-treated fruits. The fruits packaged with BNF-8% recorded a better antioxidant profile than the non-packaged fruits. Therefore, based on the results obtained from this study, the changes in the total antioxidant activity (TAA) observed in the sapota fruits relied on the packaging material. Patil et al. reported that nano-fibrillated cellulose, 1% of ginger essential oil, and green tea extract [GTE] (1%)-based active film retained the TAA of strawberry fruits [52]. Identical to our previous study, montmorillonite clay/cellulose nanocrystals from rice straw-based active films improved the storability of mango fruit and had a better TAA until the 19th day of storage [28]. In another study, cinnamon essential oil-based emulsion, bacterial CNCs amalgamated with gelatine-based films prolonged the delicious red apple storage period and restored the TAA [39]. In addition, bio-nanocomposite packaging, integrated with essential oil, prevents TAA decline by slowing down the gas exchange activity, ripening, and oxidation processes [40]. In conclusion, the BNF-8%+AO and BNF-8%+OO-treated fruits improved the storage period and maintained the TAA of the sapota fruits.

#### 3.9.4. Sensory Analysis

**Figure 11:**
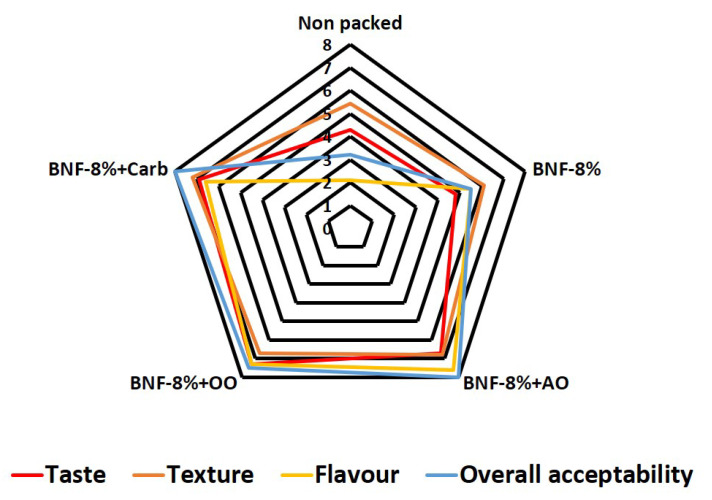
Sensory analysis of bio-nanocomposite films used for fruits package after storage.

At the end of the 12th day of storage, the sensory qualities of the treated and untreated fruits were evaluated in terms of the following criteria: flavor, texture, taste, and overall acceptability. After the storage periods, the packages were kept open for the EO vapors to escape. The sensory characteristics of BNF 8%+OO, BNF 8%+AO, and BNF 8%+carbendazim showed a better acceptability rate compared with the BNF 8% and non-packaged sapota fruit, as shown in Figure 11. However, the BNF 8% packaged and non-packaged fruits recorded the lowest sensory score, but the sensory score for the BNF 8% was better than that of the non-packaged fruits. In addition, the bio-nanocomposite packaging, integrated with essential oil, such as lavender, clove, sage, and thyme on strawberries, tomato, citrus, and table grapes, did not affect the sensory characteristics [53,54,55,56,57,58,59,60,61,62].

## 4. Conclusions

In conclusion, sapota fruits packaged using BNF-8%, BNF-8%+AO, BNF-8%+OO, BNF-8%+CARB, and non-packaged sapota fruits were investigated. The current study has proved that the BNF packaging with AO and OO vapors enhanced the shelf life of sapota fruits and reduced the postharvest decay index, identical to the BNF-8%+CARB-treated fruits. In addition, the BNF containing AO and OO vapors retained the overall fruit quality compared with the non-packaged and the BNF alone packaged sapota fruits. The BNF-8%+AO, BNF-8%+OO, and BNF-8%+CARB extended the shelf life of the sapota fruits up to 12 days. The most promising results emanating from this study were observed with the BNF-8%+AO, and BNF-8+OO, which improved the shelf life of the sapota fruits that were equivalent to the BNF-8%+CARB packaged fruits. Thus, the findings suggest that BNF-8%+OO and BNF-8%+AO vapors have the potential to be employed as natural fungicides to preserve the postharvest attributes of sapota fruits by extending their shelf life.

## Data Availability

Not applicable.

## Supplementary Materials

The following supporting information can be downloaded at: https://www.mdpi.com/article/10.3390/polym15173589/s1, Figure S1: Two dimensional AFM topographic pictures of films.
