# Supplementary figures and images for: Fabrication of Bio-Nanocomposite Packaging Films with PVA, MMt Clay Nanoparticles, CNCs, and Essential Oils for the Postharvest Preservation of Sapota Fruits
Source: Polymers (Basel). 2023 Aug 29;15(17):3589. doi: 10.3390/polym15173589 (PMC10490128; doi:10.3390/polym15173589)

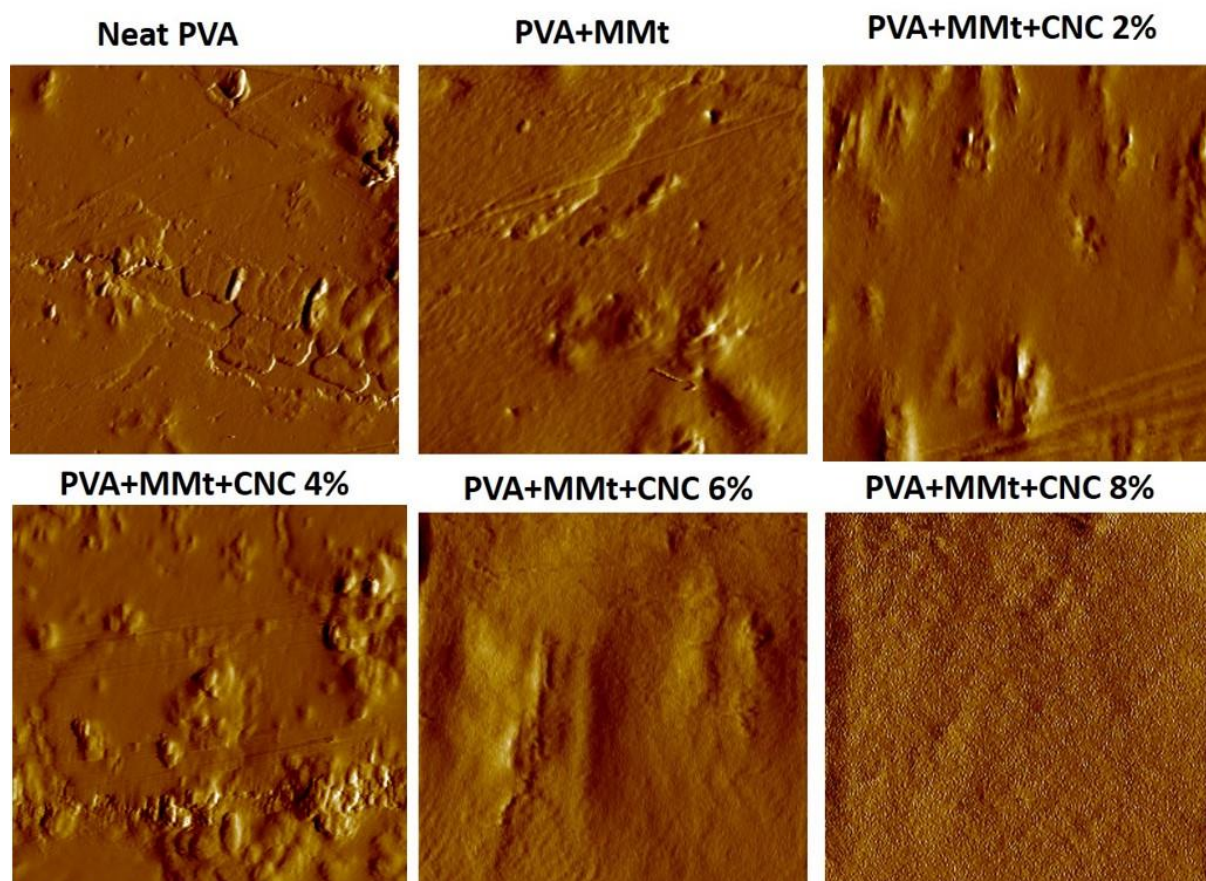

**Figure S1:** Two dimensional AFM topographic pictures of films

Supplement: Supplementary file 1 [file polymers-15-03589-s001.zip › polymers-2471825-supplementary.pdf]
